# Understanding and acceptability by Hispanic consumers of four front-of-pack food labels

**DOI:** 10.1186/s12966-017-0482-2

**Published:** 2017-03-07

**Authors:** Vanessa De la Cruz-Góngora, Pilar Torres, Alejandra Contreras-Manzano, Alejandra Jáuregui de la Mota, Verónica Mundo-Rosas, Salvador Villalpando, Guadalupe Rodríguez-Oliveros

**Affiliations:** 1 Centre for Nutrition and Health Research, National Institute of Public Health, Av. Universidad # 655, Col. Sta. Ma. Ahuacatitlán, Cuernavaca, Morelos C.P. 62100 Mexico; 2 Centre for Health Systems Research, National Institute of Public Health, Av. Universidad # 655, Col. Sta. Ma. Ahuacatitlán, Cuernavaca, Morelos C.P. 62100 Mexico; 3 Centre for Population Health Research, National Institute of Public Health, Av. Universidad # 655, Col. Sta. Ma. Ahuacatitlán, Cuernavaca, Morelos C.P. 62100 Mexico

**Keywords:** FOPL understanding, FOPL acceptability, Food labeling, Front-of-pack labels, Logos, Stars rating, Guideline daily allowance, Multiple traffic lights, Packaged foods, Hispanics

## Abstract

**Background:**

Front-of-pack food labels (FOPL) can help consumers make healthy and informed food choices. FOPL are used in the food market but evaluations of their understanding and acceptability are scanty. This study aimed to explore the subjective understanding and acceptability of four FOPL among Hispanic consumers.

**Methods:**

A qualitative study was conducted in six States of Mexico, in 18 urban elementary schools. A purposive sample of 135 parents of fifth-grade children was selected. Four FOPL were assessed: Logos, Rating Stars, Guideline Daily Allowances (GDA’s), and Multiple Traffic Lights (MTL). Trained interviewers performed 18 focus groups with the participants, using an interview guide. Participants were asked about their subjective understanding and acceptability of the FOPL, displaying 16 generic breakfast cereal boxes designed for this study (four for each FOPL), varying in their nutritional value. Afterwards, participants were asked to choose among the four cereal boxes the one to best communicate the product healthiness and their reasons for choice, proposals for improving the FOPL, and desirable characteristics for new FOPL. Finally, a socio-demographic questionnaire was applied. Thematic analysis of the transcriptions of the focus groups was performed, using Altlas.tiV5 software.

**Results:**

Logos were perceived as easy to understand, highly acceptable, and useful for decision-making; institutional endorsement of Logos was related to greater confidence in the label. The GDA’s were hard to understand considering the nutritional knowledge and time needed for interpretation. The Rating Stars were related to the quality in businesses rather than foods. The MTL were viewed as indicating the high/low content of specific nutrients, but the meaning of the amber color was not fully understood. Participants highlighted the need for a simple FOPL that allows easily identification of healthy products while considering food purchasing time limitations and interpretation of food portions.

**Conclusions:**

Logos with an institutional endorsement was the best understood and accepted FOPL, and the GDA’s and Rating Stars were the least. Our findings provide valuable insights about Hispanic consumers´ perceptions regarding FOPL and to guide public health policy. Further studies are needed in populations with chronic diseases and diverse social contexts.

## Background

The prevalence of chronic conditions related to diet, such as cardiovascular disease, cancer, and diabetes, is increasing in Hispanic consumers. Mexico has the highest prevalence of obesity -in all age groups- in both Latin America and worldwide [1]. To tackle this problem, since 2010 the Ministry of Health of Mexico announced the implementation of a useful and easy to comprehend front-of-pack food labeling (FOPL) [2]. FOPL is considered an effective strategy in helping consumers make healthier choices and informed food purchases when the FOPL is accepted and understood by the target population [3–5].

Several variations of FOPL are currently used in the food market worldwide [6, 7]. They can be classified either as nutrient-specific or summary FOPL labels [6, 8–12]. Nutrient specific labels, such as the Guideline Daily Allowances (GDA’s) and the Multiple Traffic Lights (MTL), provide nutritional information on several nutrients [6, 8, 10]. Summary labels provide information about the overall product healthiness by using nutrient profiling systems [6], they can be classified into simple and graded formats. Simple formats, such as Logos (i.e., The Pick the Tick©), are displayed only on products with relatively healthy nutrient composition [12, 13]. Graded formats, such as the Guiding Stars or the Health Star Rating system, display a ranking of stars or ticks in all packaged food products. According to these formats, more stars or ticks indicate that the food product is healthier [6, 13, 14].

Efforts to implement FOPL in Latin American countries have recently emerged. In 2012, Chile approved a warning nutrient-specific FOPL which consists on a black octagonal Logo with the expression “high in” calories, sugar, sodium, and/or saturated fat [15]. This Logo is being used on those packaged foods that exceed the nutritional limits established by the Chilean government. More recently, Ecuador adopted the MTL as a mandatory FOPL [8]. Similarly, in Mexico, the GDA’s were established since 2014 as a mandatory FOPL [16, 17]. However, previous evidence suggests that consumers might not understand and use GDA’s for making healthy food choices, and that their level of understanding might vary depending on the targeted consumer groups [18–20].

Understanding and acceptability by consumers of a specific FOPL is particularly relevant, as these perceptions may influence label use, and eventually, food purchasing [3]. FOPL understanding is estimated by using either objective or subjective measures [21, 22]. Subjective understanding represents the extent to which consumers consider they have understood a label. FOPL acceptability is assessed by exploring perceptions of consumers of both liking and confidence in specific labels [22]. Qualitative research methods are particularly valuable to explore personal experiences of consumers, allowing to have in-depth insights about FOPL understanding and acceptability [23, 24].

Before determining FOPL effects on consumer purchasing behaviour, FOPL understanding and acceptability should be studied in those contexts where they are or intended to be implemented [8, 22]. For example, Latin American consumers from economic deprived areas might have lower educational levels, limited mathematical and reading skills, and different cultural norms than consumers from affluent areas of developed countries [8, 25]. These differences should be considered by decision-makers when implementing a mandatory FOPL system [8].

Studies conducted mostly in European countries suggest that the MTL is the most accepted and understood FOPL [8, 26, 27]. In this regard, international research indicates mixed results [28]. In Latin America, Gregori and collaborators explored the understanding of the GDA’s by the Chilean population [29]. They found that more than 60% of participants were not able to identify the meaning of the nutritional information provided by the GDA’s [29]. Stern and collaborators reported that only 56.3% of Mexican undergraduate nutrition students were able to correctly identify the servings per container using GDA’s [20]. A recent study exploring FOPL preferences among parents of school-aged children from Chile, Ecuador, and Argentina reported that the GDA’s were the preferred FOPL among high income participants, whereas the MTL and the black octagonal Logo used in Chile were the preferred FOPL among low income participants [8]. In Latin-American countries, including Mexico, there are limited studies documenting understanding and acceptability by consumers of currently used and new FOPL. Therefore, this study aimed to explore the subjective understanding and acceptability of four FOPL (the Logos, the Stars Rating, the GDA’s, and the MTL). Our study intended to expand current evidence for designing a FOPL targeted to Hispanic consumers, and eventually, informing the design of the mandatory FOPL currently used in Mexico.

## Methods

### Study design and sampling

A qualitative study was conducted in the southern, central, and northern regions of Mexico. Two States in each region were selected based on their socio-economic and cultural diversity within the country [30–34]. In each State, 18 urban elementary schools pertaining to the low, middle, and high socio-economic status were randomly selected from the census of elementary schools, provided by the Ministry of Education of Mexico. This federal agency classifies the socio-economic status of Mexican schools, according to an index developed at the neighborhood level. This index include information from the National census of Mexico such as availability of sewage, access to electricity and potable water, and paved streets as well as employment rate, education, and income, among other criteria.

A purposive sample of 135 parents of fifth-grade children (129 mothers and 6 fathers) was selected from the elementary schools. Mothers were identified as key informants while being traditionally responsible for making household food purchasing and feeding decisions [35, 36]. A total of 48, 58, and 38 participants were selected from low, middle, and high SES schools, respectively (Table 1). They were identified and recruited as follows.

**Table 1:** Descriptive characteristics of participants in the focus groups, stratified by socio-economic status

	Socio-economic Status			All
	Low	Middle	High	
	Frequency (%)			
Sample (n)	41	56	38	135
Sex (female)	97.5	92.8	97.3	95.6
Age (years; *media ± SD)*	34.5 ± 7.3	36 ± 6.5	37.9 ± 5.5	13.1 ± 6.6
Education Level				
None	2.3	3.6	0.0	2.2
Elementary School	14.4	21.4	0.0	13.3
Junior High School	51.1	26.8	10.4	29.6
High school/Technical	25.0	32.1	29.0	28.9
Graduate degree	7.2	16.1	55.3	24.4
Post-graduate degree	0.0	0.0	5.3	1.48
Occupation				
Housewife	83.0	64.3	68.4	71.1
Employed	12.2	25.0	29.0	22.2
Unemployed	4.8	3.6	0.0	2.9
Other	0.0	7.1	2.6	3.7
Marital status				
Single	17.1	17.8	2.6	2.2
Married	78.0	78.6	92.1	82.2
Divorced	4.9	1.8	5.3	3.7
Widow	0.0	1.8	0.0	0.7
Frequency of trips to the supermarket				
Everyday	12.3	7.1	10.6	9.6
Two to three times per week	21.9	25.0	26.3	24.4
Once a week	19.5	32.1	44.7	31.8
Every two weeks	34.1	17.9	13.1	21.5
Once a month	7.3	14.3	5.3	9.6
Doesn’t know	4.9	3.6	0.0	2.9
Estimated monthly food expenditure (Mexican pesos)				
<1000	29.3	30.4	2.7	22.2
1000 – 2499	48.7	44.6	26.3	40.7
2500 – 5000	9.8	17.8	52.6	25.2
>5000	0.0	3.6	10.5	4.4
Doesn’t know	12.2	3.6	7.9	7.4

With the willingness of the Ministry of Health of Mexico, the researchers sent a letter to the principals of the selected schools asking for their approval to conduct the study. This letter specified the study objectives, recruitment techniques, and data collection procedures. All of the contacted principals agreed to conduct the study with verbal consent. Afterwards, a memo written by the researchers was sent by the schools’ teacher to the households of fifth-grade children, inviting their mothers and other household members who are considered responsible for household food purchasing and feeding to participate in the focus groups. The memo indicated the study objectives, data collection procedures, ethical considerations, and logistic information regarding the focus groups sessions.

### FOPL evaluated

**Fig. 1:**
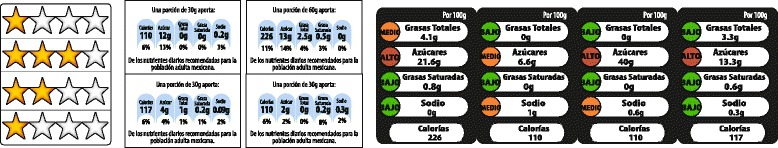
Front-of-pack labeling images. Captions: Rating Stars, GDA’s, Multiple Traffic Light

**Fig. 2:**
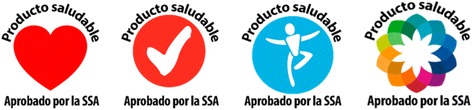
Front-of-pack Logo designs. Captions: Pick the Tick**©**, Wind Spinner**©**, Heart, Human silhouette

We evaluated the subjective understanding and acceptability of four FOPL: the Logos, the Stars Rating, the GDA’s, and the MTL (Figs. 1 and 2). We selected these labels while they represent some of the main FOPL used in the food market worldwide [6, 9, 11, 37]. Four different Logos were assessed, the Pick the Tick©, and a Wind Spinner© used in the international and local markets, respectively, as well as two generic logos designed for this study (a heart and a human silhouette). The heart Logo included design features used by some international FOPL [6, 12, 13]. The human silhouette resembled an active and normal-weight person, resembling Logos used in local breakfast cereals retailed by global food companies [6, 12, 13]. Considering that the Logos are usually endorsed by an institution, each one came with the legend “Approved by the Health Ministry” on the lower part of the image [12].

### Testing material

A total of 16 generic breakfast cereal boxes (32.0 × 19.5 × 8.0 cm) were designed, four for each FOPL. To emulate a market environment where consumers can compare similar products with different nutrient values, we selected four breakfast cereals from the local market, looking for diversity in nutritional value, in order to be ranked using the Stars Rating, the GDA’s, and the MTL. Accordingly, each of the four boxes used for each of these FOPL varied in their nutritional value (except for the Logo boxes while this FOPL is not intended to provide nutrient ranking information). The nutritional labeling of the back-of-pack panel was also displayed in each box and was consistent with the information shown in the FOPL. Considering that the Logos endorsed by Health agencies are generally placed on healthier products, the back-of pack panel of the four boxes using Logos corresponded to the “healthiest” breakfast cereal. The front label was placed in the upper right corner of the package, taking up between 8% and 20% of the front of the box. To avoid bias in the information, each box was identified by using an alphabetic code.

### Data collection

Eighteen focus groups were carried out: 6 in the northern, 5 in the central, and 7 in the southern region. A total of 135 participants attended to the focus groups, having eight participants on average per session. Sessions were conducted at the school in a private place such as the classroom or the auditorium, without the presence of the teaching staff or of the children of the participants.

A focus group guide was developed including the following subjects of inquiry regarding FOPL perceptions: 1) FOPL understanding explored through what is understood and what is not understood (i.e., design features and numeric information), 2) FOPL acceptability explored through liking and confidence, 3) The FOLP that from the participants perspective had ability to best communicate the product healthiness and, 4) Proposals for improving the evaluated FOPL and desirable characteristics of a new FOPL. At the end of the focus groups, socio-demographic information such as age, sex, marital status, educational attainment, occupation, frequent food purchases in the supermarket, and estimated monthly food expenditure, was collected using a self-administered questionnaire.

Focus groups were held in school facilities and led by trained personnel. The focus group session was led by a psychologist, supported by a nutritionist, physician, or nurse. The focus groups were audio-recorded with prior informed consent of the participants, and were further transcribed verbatim. Sessions had an average length of 1.15 h.

The dynamics for each focus group initiated by asking the participants if they are usually responsible for making household food purchasing and feeding decisions. Afterwards, participants were asked about how they describe a healthy and unhealthy packaged food. Afterwards, a choice exercise was conducted with the participants showing the four boxes of each FOPL, in the following order: 1) Logos, 2) Stars Rating, 3) GDA’s, and 4) MTL. The same presentation order was followed in all focus groups. For each FOPL, participants were asked to explore individually each of the four cereal boxes with different nutritional value. The participants were asked about their perceptions regarding subjective understanding and acceptability of the evaluated FOPL. As a final step, a set of four cereal boxes was displayed to the participants, one for each FOPL with the healthiest nutrition profile. In this step, participants were asked to compare each FOPL and choose the one they perceived had the ability to best communicate the product healthiness and to express the reasons of their choice. As complementary information, participants were encouraged to discuss additional issues regarding FOPL understanding and acceptability, to provide feedback for improving the evaluated FOPL, and expressing desirable characteristics of new FOPL.

### Data analysis

Thematic analysis of the transcriptions of the focus groups was performed to identify themes which were created by identifying patterns in data that made a meaningful contribution to our understanding of the study aims [38, 39]. Thematic analysis was conducted through the process of coding in six phases: familiarization with data, generating initial codes, searching for themes among codes, reviewing themes, defining and naming themes, and producing the final report, as following described.

First, we reviewed the transcriptions for initial exploration of the data. Second, we generated initial or *a priori* general codes based on the performed literature review and the study aims. That is, an analysis that uses literature as a foreground to create pre-existing codes of exploration [21, 40]. Third, a more deductive process using these *a priori* codes was combined with inductive reflection on codes which emerged during fieldwork and subsequent reading of data. Both the *a priori* and emergent codes were used to identify patterns and themes in the discourse (segments of transcriptions). These themes are based on manifested social meanings and constructions, and reveal explicit social perceptions around the research topic [39, 41].

A code book composed by main themes or categories and codes was developed. Using Altlas.ti V5 software [42], all transcriptions were coded by four researchers. Inter-encoder standardization exercises were carried out. We coded perceptions expressed by most of the participants, and also those expressed by a few of them, as well as perceptions not directly related to the study aims, in order to include less common or divergent voices or statements.

Afterwards, we developed thematic matrixes [42] according to the following main themes: 1) Understanding and not understanding FOPL, 2) FOPL acceptability (liking it and confidence in it), 3) The FOPL that from the participants perspective had the ability to best communicate the product healthiness, and 4) Proposals of the participants for improving the evaluated FOPL. The matrixes were organized by both SES level and geographical region which allowed the comparison of the coded text segments by each of the main themes. The accuracy of the data analysis was confirmed using the interpretative triangulation technique [43], in which two researchers analyzed the same data individually, each reaching consistent results.

### Ethical considerations

The parents participated voluntarily and provided oral informed consent, including authorization to record the focus group sessions. They did not receive monetary incentives for attending the focus group. They were informed that their identity and information provided would be anonymous and that rejecting to participate would not cause them any inconveniences for themselves or their children at the school. They were also informed that they could stop participating in the study at any time. A form with the contact information of the principal investigator and the Ethics Committee representative of the National Institute of Public Health of Mexico (INSP) was provided to participants. The Ethics Review Board and the Research Commission of the INSP approved the research protocol.

## Results

### Characteristics of the participants

Table 1 shows the socio-demographic characteristics of the study participants; those from high SES were on average older (37.9 years old), more educated (bachelor’s degree or higher), and with higher monthly food expenditure (> $2 500.00 Mexican pesos/$147.00 US dollars), compared to those with a low SES.

### Subjective understanding and acceptability of FOPL

Reported perceptions on subjective understanding and acceptability were consistent across SES, mainly lexical differences were observed between participants from different SES levels. These differences were mostly related with a more extensive vocabulary among participants from the high SES (i.e., use of nutrition technical terms). Consistently, the results are presented regardless of SES or other socio-demographic characteristics, while the SES and geographical region are indicated at the end of each quotation.

Regarding the perceptions of participants about the healthiness of food packaged products, most of them indicated that a healthy food contains “low sugar and fats”, while a few of them added that “integral food products” are healthy. In contrast, participants referred to unhealthy products as those that contain artificial flavours (i.e., chocolate flavour), colorants, and food preservatives.

### The logos

Participants agree that Logos are “highly acceptable and useful for decision-making during food purchases”. Participants explain that Logos are easy to understand because “everything can be instantly read and they have a visual element that is easy to catch at a glance”:

*Participant (P1): “I’m not the type [of person] that reads everything, like the lady said, because of lack time, we only go there [*the supermarket*] and grab things [*packaged products*], but this Logo would be good because it makes it easier.”*

*Moderator (M): “¿How hard or how easy is it to understand this type of front-of-pack labeling* [Logos]*?”*

*P1: “Easy, it’s simple because contents are displayed there, you just read it and that’s it!”*

*P2: “Well, the visual impact is quick because you instantly read everything”. (Middle SES, central region)*

Consumers consistently express that the Logos are useful at the moment of making a purchase decision because it means that “the product is good for their health and their families”. Participants indicate that Logos helped choosing one product over another because “they are endorsed by the Ministry of Health” -The highest health authority in Mexico-. Participants expressed feeling a “high degree of confidence” in the legend of the Ministry of Health located alongside each Logo. Most of the participants thought that the acronym of the Ministry of Health “*SSA*” (in Spanish) is “trustworthy”, because it represents the endorsement of a respectable institution that would not falsify information on the nutritional quality of the product, and that guards the health of the population. Thus, by having the approval of this institution, the products “guarantee” the health of their family and children:

*P1: “Well, this* [Logo] *has been approved by the Ministry of Health, hasn’t it?”*

*P2: “I believe that by putting this* [Logo] *that shows that it has gone through a registration process, a quality process, and all of that* [so the product] *is healthy and good for its consumption, I mean, for the family in general.” (High SES, southern region).*

*M: “What message does this type of [Logos]… give you?”*

*P1: “Confidence in that what one is purchasing is Ok; it’s already been evaluated, it’s guaranteed, as they [the Ministry of Health] say.” (Middle SES, northern region)*

*P1: “Well yes… the Ministry of Health, they cannot lie to us […] about our health.” (Middle SES, central region)*

Some participants expressed specific perceptions about the four evaluated Logos that were the Pick the Tick©, Wind Spinner©, heart, and human silhouette. In regards to the Pick the Tick©, a few participants consider that this Logo indicate that “the food product is good”, but that they do not fully understand the nutritional characteristics that this FOPL intended to inform. In regards to the Wind Spinner©, some participants agree that they like the design while it resembles a “flower”. A few of them also indicate they do not understand the meaning of this Logo in regards to the perceived healthiness or the nutritional value the food product. Most of the participants indicated that the heart and human silhouette Logos were understood as indicators that the product “was good for the heart” and “for maintaining a stable weight”, respectively. Three participants related the heart Logo with a hearth image used by a cooking oil brand retailed at the local market, claiming that the product having this Logo “is good for the heart and can be consumed without health concerns”. A few participants considered that the human silhouette Logo represented a “thin person”, and therefore the food product having this Logo is either “good for athletes or helpful to reduce weight”. A few of them also highlighted that “they do not fully understand the message transmitted by the human silhouette”.

### Rating stars

There is an agreement among participants from the different SES that Rating Stars are barely useful, while being perceived as a “very commercial and overused indicator” to show the quality of hotels and restaurants. This FOPL was considered “confusing and hardly serious” for evaluating the product healthiness. Most participants understood the connection between the food quality and the number of stars, indicating that “the greater the number of stars, the greater the quality of the product”. Moreover, some participants expressed that despite being able to understand this relation, the parameters used to assign the stars were not clear. Thus, they did not relate the number of stars with the product healthiness and claimed that “its nutritional understanding was of little use when choosing a food product”:

*P1: “… I don’t understand it, the stars, I don’t understand what they mean… It does not give a message. What do we understand? That the more stars the higher quality?” (Middle SES, southern region)*

*P1: “But I believe that it’s marketing [of the Rating Stars], is what visually draws your attention. Of course, you would have to see what the stars are grading. Maybe the one with the most stars is the worst!” (High SES, southern region)*

*P1: “I didn’t like the stars eh, because I consider that there are concepts to relate some things, and the stars are already very, very overused, you have them, overused in movies.” (High SES, northern region)*

*M: “What do more stars mean to you?”*

*P1: “[…] if there are five stars (laughs), the better, right?”*

*P2: “What happens is that we would also have to know the parameters being used when grading with the stars.”*

*P3: “Yes, in the same way, that would also be the meaning of why four stars or why three, that would also be the same.”*

*P4: “Well yes, if I found this box, I would need an instruction, explaining, what do the stars mean?” (Low SES, central region)*

A few participants claimed liking the Rating Stars because they associated a quality-cost relationship with this FOPL. They explained that when considering the cost, the Rating Stars would allow them to choose the product of “the best quality” according to their budget, as observed in the following statement:

*P1: “To me it* [The Rating Stars] *also seems more… more adaptable let’s say, because with the other one [Logo], it’s “yes or no” and that’s it, so maybe we don’t have enough money to buy the “yes” option and we have to stick with the “no”; instead with this one* [The Rating Stars]*, we have the option of saying well I want the “yes”, but because of my budget I’ll adapt to the “middle yes”, and then the “middle yes” is better than the “no”, so it feels more adaptable to me and easier to handle.” (Middle SES, northern region)*

### The GDA’s

Most participants stated “not to like, nor perceive the usefulness of the GDA’s”, because of the lack of understanding of technical terms, the arithmetic procedure needed to identify the serving size equivalent, the time needed to analyze the elements displayed, and the overall lack of comprehension of the nutrition information provided:

*P2: “I also say that for most Mexicans, this* [the GDA’s] *doesn’t mean anything, there are a lot of people who do not have, including myself, the training; even though, one has tried and more or less knows, but most people do not know, they do not see or read this.” (High SES, northern region)*

*P1: “We don’t have the knowledge.”*

*P2: “I like it but the thing is…”*

*P3: “We don’t know.” (High SES, southern region)*

*P1: “Why should I complicate my life again by having to multiply?” (Middle SES, northern region)*

*P1: “No one agrees with this one* [GDA’s]*, nobody.”*

*M: “Did anybody like this one?”*

*P9: “No one.” (Low SES, southern region)*

Several participants declared that the GDA’s do not allow them to judge if the cereal product was “good” or “bad” for their health and some of them indicated that the information provided by the GDA’s “repeats the nutritional labeling on the back-of-pack and is confusing”:

*P3: “It’s the same as the one on the back* [back-of-pack Nutrient Facts panel]”

*P4: “We go back to the same here, it’s math!”*

*P5: “To me, this type of system feels complicated, I mean, you won’t stop to examine and read it and to verify if the information matches the one on the nutritional table, we want something more agile!” (Middle SES, northern region)*

A minority of the participants expressed confidence in the GDA’s, stating that they “offered more information on the product”. Some indicated that this FOPL was of their liking, arguing mostly that “it provided more information and was more visible” by being placed at the front of the pack.

### Multiple traffic lights

At first, the participants expressed that “they liked and appreciated this system”, mostly because they could identify the colors of the labeling. They relate them with the traffic light colors, specifically with a scale of “high/middle/low content” of some of the nutrients of the product. They agreed that the traffic light colors were easy to understand individually. Some participants pointed out that they liked the traffic light because “it was easy to understand and therefore, useful when choosing food products”. As for the understanding of each color, all participants easily identified the green and red colors as “low/good and high/bad” indicators for the product quality. In contrast, participants were indecisive about the meaning of the amber color:

*P1: “Besides, we are very visual; many times you guide yourself through colors, it happens in your day to day experience, traffic lights for example… this is good for me, but the one with four reds, well, you don’t even consider it, you don’t even get close to it.” (High SES, central region)*

*P1: “Hmm, the green one, I would classify it like the colors from traffic light, right?”*

*P2: “Green means that you can consume this product because it is giving you the go ahead that you can consume it. The red says you can’t consume this* [product], *and the yellow, well, it is half way there from yes.” (Middle SES, central region).*

After comparing the four MTL cereal boxes, regardless of SES level, most participants found the combination of colors “confusing”. Participants referred that the MTL was difficult to understand, especially when deciding which product to choose. They commented that “making food choices would be easier if the traffic lights provided a clear contrast” (i.e., all red or green), when using this FOPL in similar products.

Participants expressed having low acceptability for the MTL because “it does not inspire confidence, they do not like the design, and the information seems incomplete and repetitive”. There were heterogeneous opinions from the participants on whether the MTL informs if the product is healthy or not. The contrasts of the MTL colors in the labeling were easily identifiable (i.e., all red or green). Intermediate variations caused difficulty when choosing the healthiest product, so the participants considered MTL “not useful” for this purpose. Likewise, they recommended grading the product with only one color (red, green, or amber), and not grading the individual nutrients:

*P2: “Yes because if you have the 3 colors, you say this is good, this is medium and this one is bad so, you have those 3 things…”*

*P3: “And then, if we are wrong? [in choosing]” (Low SES, northern region)*

*P1: “I usually go to the supermarket and choose quickly ´this one yes this one no´. I even got more confused when I saw green, red, and yellow, like what’s that about?”*

*P2: “It’s confusing; I would be indecisive with it.” (High SES, northern region)*

*M: “How would it [the MTL] have to be for you to say, ah this one is healthy, and I’ll take it?”*

*P1: “That it has all green colors.”*

*P2: “Preferably just green color.” (High SES; central region)*

### The FOPL that best communicate product healthiness

In the final step of the focus group choice exercise, the participants agreed that the FOPL that had the ability to best communicate the product healthiness was the Logo, followed by the MTL, Rating Stars, and finally, the GDA’s. The main reasons for selecting the Logo were consistent with the perceptions reported above, related with its easy and clear interpretation as “a simple indicator that the product is healthy”, and its potential usefulness for food purchasing decision-making. Participants also expressed feeling confidence or trustworthiness associated with the endorsement of the Ministry of Health of Mexico shown in the Logo boxes. In contrast, the GDA’s were considered the least consumer-friendly FOPL because the use of technical terms, the time needed to analyze the quantitative elements displayed, and the overall lack of understanding of the nutritional information provided by this FOPL, as observed in the following statements:

*M: “Which label would you think has the least ability to communicate the product healthiness?”*

*P1: “The Z [GDA's identifier], because it comes in grams and percentages, I do not understand it, I do not understand if the product is healthy or not.” (Low SES, central region)*

*M: “From these four labels which one do you think has the best and the least ability to communicate the product healthiness?”*

*P1: “[The least is] the percentage [GDA's], it is very difficult and I do not understand the scale.”*

*P2: “The Z cereal box, I think the same [than P1], I choose it [GDA's] for the same reason.”*

*P3: “The Z box [GDA's]." (Medium SES, northern region)*

### Proposals of the participants

Throughout focus groups participants consistently and repetitively called on nutrition experts, suggesting the need for a simple labeling that accurately identifies healthier products, and informs in a simple way “which are the products that are good for one’s health”. Participants agreed that a clear and precise recommendation on the nutritional quality of the product would be a viable option to orient consumption by considering the lack of time they have when shopping, as well as the general inability of people to interpret the information about the food ingredients and serving sizes. They also stated they would rather have the Ministry of Health tell them which product they should choose, based on the awarding of a stamp or endorsement, and also to inform on the characteristics for which the stamp was awarded.

## Discussion

This study shows that the Logos were the best understood and accepted FOPL by the participants (Hispanic adult consumers), while the Guideline Daily Allowances and the Rating Stars were the least understood and accepted FOPL. The endorsement from the Ministry of Health was considered a source of trustworthiness about the perceived product healthiness.

As shown in previous studies conducted in developed countries [21, 40], perceived advantages of the Logos found in our study relate with their easy and quick identification, not needing to do mathematical calculations or having technical knowledge to identify the nutritional qualities of the product. Experiences with the use of Logos designed as public health strategies, such as the Pick the Tick© [12], the Green Keyhole© [44], and the Heart symbol [45], among others, have shown that this FOPL allows consumers to make healthy choices of products that are low in sodium, fat, or high in fiber, in an easy and simple way [6]. Some studies have shown positive impacts in the selection of healthy products by consumers when implementing a mandatory, easy to understand, and acceptable FOPL [6, 21]. For example, Larsson et al., documented that the ingestion of low fat food products was higher in consumers with knowledge of the Green Keyhole© [44]. In Chile and Ecuador, efforts have been made to develop new FOPL according to consumers’ preferences; nevertheless, the effectiveness of those FOPL have not been evaluated [6]. Future studies should explore the impact of these newly developed FOPL on the food purchasing behaviour of consumers.

Experiences with the Traffic Light plus Overall Rating, a mixed FOPL combining color coded information by rating both individual nutrients and whole foods while including an interpretive element, have shown to be effective for assisting consumers to identify healthier products [46]. Our results suggest that the design of the Logo may influence these outcomes (i.e., the hearth and the human silhouette were both the most understood Logos). Consistently, some authors have shown that FOPL motivation to use, understanding and effectiveness depends on the FOPL design preferences of the target population [47, 48].

The MTL was the second most understood and accepted FOPL across SES levels. This is consistent with other studies, showing higher levels of understanding and acceptability across income groups, for both the Simple Traffic Light and the MTL [49, 50]. Research on European consumers has demonstrated that they prefer simplified FOPL and that they understand the MTL formats [3, 50]; they also needed significantly less time to evaluate simpler FOPL compared to the more complex labeling formats [51].

The MTL was accepted by most participants despite its use of technical terms, however, the confusion generated by the color variation and lack of predominance of a color diminished the usefulness of this FOPL in helping participants’ intention to make a “healthy” choice. Hawley et al., [19], documented the results of a review of research studies on consumer understanding and behaviours relating to front-of-pack labeling. Their results indicated that the MTL system has most consistently helped consumers from industrialized countries to identify healthier products [19]. However, there is little evidence regarding developing countries.

The least understood and accepted nutritional FOPL were the Rating Stars and the GDA’s. The first, was not identified as a nutritional indicator of the quality of food products; instead, it was associated with quality indicators used in other commercial sectors such as hotels, the film industry, and restaurants. Using a FOPL transmitting a wrong message (i.e., associated to other characteristics rather than nutritional quality) may create mistrust in FOPL, instead of helping to make a right and informed food choice. In this sense, the roll out of most FOPL is generally supported by social marketing, communication, and/or education strategies, which may help ameliorate consumers’ mistrust and increase potential FOPL effectiveness [52]. Additionally, formative research may inform the design of appropriate communication messages used by such strategies [18, 53, 54].

The GDA’s were neither understood nor accepted by most of the participants. They were not deemed comprehensible or useful for “easily” transmitting the product healthiness. First, they required people to visualize the serving size, and then, to perform mathematical operations in order to incorporate the meaning of the percentages with respect to the recommended daily intake. Our findings are in line with those of a study conducted among Mexican nutrition students who performed incorrect mathematical calculations, and were confused by the serving size declared in the GDA’s [20]. At the time of this research, most global food companies in Mexico used GDA’s voluntarily. Currently, the use of GDA’s is a mandatory FOPL supported by the Ministry of Health [16]. However, our findings indicate that this FOPL does not fully allow consumers to judge the perceived healthiness of food products, including those who declared liking it.

Awarding a stamp or endorsement from the Ministry of Health rendered confidence in the label. Although this finding had not been previously documented in Mexico, it has been shown that endorsement may positively influence the perceived quality of products by consumers [51, 55].

The SES and educational attainment of the participants in our sample did not appear to influence the understanding of MTL and GDA’s. This is contrasting with other studies from developed countries showing that the education level influences the understanding or acceptability of FOPL [3, 56].

In this study, the participants might have not considered relevant factors such as the brand and the price of the food product, when evaluating the FOP. While this study did not intend to evaluate FOPL effectiveness, further studies performed in real purchasing settings might be valuable for understanding these factors. Similarly, having the endorsement of the Ministry of Health on the Logos may have increased the acceptability of this FOPL. Nevertheless, we consider that the subjective understanding of this FOPL might be barely influenced by the institutional endorsement. We acknowledge potential ordering effects in the perceptions expressed by participants, while the order in which FOPL were assessed did not vary across focus groups. Aiming to reduce this potential limitation of our study, the four boxes with the evaluated FOPL were shown simultaneously to participants before finishing the focus groups, allowing them to compare the evaluated FOPL and expand or modify already expressed statements.

To our knowledge, this is the first qualitative study that explores the subjective understanding and acceptability of four internationally used FOPL among Mexican population. Accordingly, our study contributes to the field of nutrition by informing about behavioural aspects of Hispanic consumers in regards to their understanding and acceptability of different FOPL. Another strength of our study, is the inclusion of participants from diverse geographical regions and different SES. This allowed us to explore a broader set of perspectives from parents with different educational attainment and cultural background. We studied a specific group of Mexican consumers whose opinions might differ from those of the general population. While generalization of current results might be considered cautiously, the manifested social meanings and explicit social perceptions documented in this study are relevant for the design of FOPL targeted to Hispanic populations, where mothers are involved in making household purchasing and feeding decisions [35, 36].

## Conclusions

Logos with an institutional endorsement were the most accepted and better understood FOPL by the studied Hispanic adult consumers, considering their perceived easy and quick identification. The least understood and accepted FOPL were the GDA’s and the Rating Stars. The GDA’s were perceived as complex FOPL, while the Rating Stars were considered not fully suitable as a FOPL because they are generally used to evaluate quality features non-related with nutrition. MTL were fairly accepted; nevertheless, the meaning of the amber color used in this FOPL was hard to understand.

Future research should explore the effectiveness of the evaluated or new FOPL formats for informing about the overall nutrient profile of food products among Hispanic consumers. Evidence is also needed to explore how FOPL is understood and used in real-world settings, what is their effect on retailer sales, and how the continued use of FOPL would impact on consumers’ dietary patterns, nutritional status and health. Similarly, further studies must be conducted in populations living in developing countries and in those with chronic health conditions, such as diabetes and hypertension.

FOPL is a strategy that can assist consumers in making healthy food choices and informed purchases. To promote healthy consumption patterns, complementary population-wide strategies such as food reformulation and behaviour change interventions, are required in line with updated and systems-oriented public health policies to address the global burden of diet-related chronic diseases.

## Abbreviations

FOPL: Front-of-pack food labels
GDA’s: Guideline daily allowance
MTL: Multiple traffic lights
SSA: Ministry of health of mexico (Spanish acronym)
